# The Contribution of Genetic Diversity to Subdivide Populations Living in the Silk Road of China

**DOI:** 10.1371/journal.pone.0097344

**Published:** 2014-05-14

**Authors:** Zhe Zhang, Shuguang Wei, Hongsheng Gui, Zuyi Yuan, Shengbin Li

**Affiliations:** 1 Key Laboratory of Environment and Gene Related to Diseases, Ministry of Education, College of Medicine, Xi'an Jiaotong University, Shaanxi, China; 2 The Key Laboratory of National Ministry of Health for Forensic Sciences, College of Medicine and Forensics, Xi'an Jiaotong University, Shaanxi, China; University of Utah, United States of America

## Abstract

There are several indigenous ethnic populations along the silk road in the Northwest of China that display clear differences in culture and social customs, perhaps as a result of geographic isolation and different linguistic traditions. However, extensive trade and other interactions probably facilitated the admixture of different gene pools between these populations over the last two millennia. To further explore the evolutionary relationships of the 13 ethnic populations residing in Northwest China and to reveal the features of population admixture, the 9 most-commonly employed CODIS loci (D3S1358, TH01, D5S818, D13S317, D7S820, CSF1PO, vWA, TPOX, FGA) were selected for genotyping and further analysis. Phylogenetic tree and principal component analysis revealed clear pattern of population differentiation between 4 populations living in Sinkiang Uighur Autonomous Region and other 9 populations dwelled in the upper regions of Silk Road. R matrix regression showed high-level gene flow and population admixture dose exist among these ethic populations in the Northwest region of China. Furthermore, the Mantel test suggests that larger percent of genetic variance (21.58% versus 2.3%) can be explained by geographic isolation than linguistic barriers, which matched with the contribution of geographic factors to other world populations.

## Introduction

**Figure 1:**
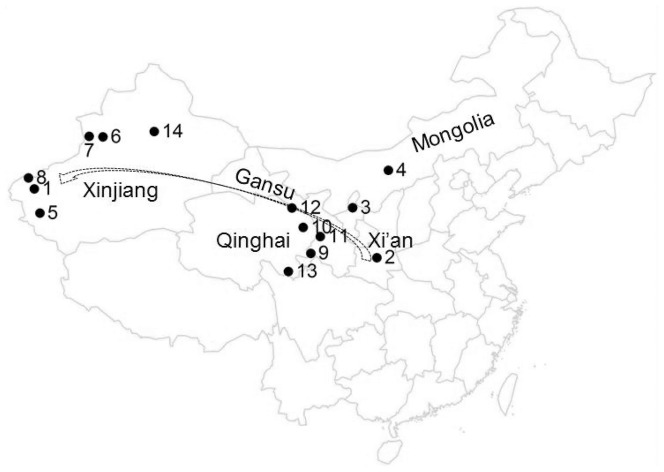
Geography of the studied region. Note: 1, Kashgar; 2, Han_XA; 3, Hui; 4, Mongol; 5, Uyghur; 6, Kazakh; 7, Uzbek; 8, Kirghiz; 9, Salar; 10, Tu; 11, Dongxiang; 12, Yugur; 13, Baoan; 14, Han_XJ. The dash line with arrow is the Silk Road within China; from Xi'an to Kashgar and beyond. The numbers from 2 to 14 are the locations of 13 populations in this study.

The Northwest region of China has a very complex geography, encompassing mountains, plateaus and basins, as well as some special landscapes, such as the Gobi desert. There are at least 20 ethnic populations and isolated groups that reside in this region. The Han, Hui and Mongolian people are three of the largest ethnic groups in China. The Han ethnic group has a population of more than 1 billion. The Hui and Mongolian ethnic groups each have populations of more than 5 million and are regarded as typical examples of Chinese ethnic minorities [1]. Of the populations that live in Sinkiang Uighur Autonomous Region, all are aboriginals except for the Han ethnic group, which has migrated to the region from Central China since the 1950s. According to written records, the Uyghur ethnic group has been in frequent contact with both eastern and western populations since the 3rd Century B.C. The immigration of the Uzbek, Kazakh and Kirghiz ethnic groups was the result of the expansion of Mongol Empire in the 13th century, and their ancestors may be the people that inhabited central Asia 2,000 years ago. Of the five ethnic populations living in the Qinghai and Gansu provinces, the Yugur ethnic population has a relatively long history. The other four populations are most probably the product of population admixtures among the Mongolian, Hui, Han and Tibetan ethnic populations [2]–[4] (Figure 1).

The “Silk Road”, which could date back to the Western Han Dynasty, starts geographically from the ancient capital Chang-an, passes through the “Hosi Corridor” and Sinkiang Uighur Autonomous Region and extends into Central Asia, India and finally the Mediterranean region. Previous research has suggested that extensive genetic admixture exists in the Silk Road region [5], [6]. Evidence from the mitochondrial hyper-variable region showed that populations in central Asia contain gene pool elements of both Eastern and Western Euro-Asians. Furthermore, historical records indicate that factors such as religious belief, marriage customs, linguistic traditions and migratory history may have played important roles in shaping the matrilineal genetic structure of the populations living in this region [7]. However, these investigations have seldom examined the genetic structure and population differentiation of the populations living near the starting point of the Silk Road.

Addressing major issues in the field of human genetics requires multiple types of genetic markers and various analytical methods and statistical models and the consideration of geographic, linguistic and social factors [8]. In recent years, several Chinese investigators have examined population differentiation and admixture patterns for Chinese populations and some Central Asian populations. On the basis of the allele frequency data of 15∼30 STR loci, Chu et al. constructed a phylogenetic tree for 32 East Asian populations and proposed a hypothesis for the origin of East Asian people [9]. Using Y haplotype features, Su and Jin inferred the origin of the Chinese Han population and East Asian people and hypothesized that the Northern Chinese Han population derives from migrants from the Southern Chinese Han population [10]. Recently, Xie et al. analyzed Y chromosome STRs and SNPs from selected individuals living in Gansu Province and suggested that they might be the offspring of ancient Roman soldiers [11]. Moreover, Zhang et al. have used mitochondrial sequence diversity to study the evolution and origin of Chinese populations. They constructed a phylogenetic tree for the Chinese Han populationbased on mitochondrial haplogroups that has been widely employed in later investigations on mitochondrial polymorphisms in East Asian populations [12]. Most of the previous studies agree that high genetic differentiation exists among Chinese populations and that the gene flow and genetic admixture are very complex. Samples covering a wider range and larger size are needed to improve the robustness of the statistical analysis, and more sophisticated statistical models and analysis should also render the results more convincing.

Genetic markers on the Y chromosome and on mitochondrial DNA, such as Y-STRs, Y-SNPs and mitochondrial hyper-variable regions I and II, have low recombination rates and lack of recombination respectively, are widely used to address the genetic differentiation between populations[13], [14]. Confounding issues such as low effective sample size and ascertainment bias can be problematic, and genetic markers on the Y chromosome are especially susceptible to genetic drift and male reproductive functions[10], [15]. Microsatellites have been applied in detecting human genome variation, conducting linkage analysis and in forensic applications,such as DNA fingerprinting. Microsatellites have proven to be especially useful in studies of the evolutionary relationships between species or between populations with relatively close genetic relationships [16]. These studies suggest that the behavior of autosomal genetic markers is similar to human linguistic patterns [17].

In this paper, we have selected 13 representative populations (12 different ethnic groups) living in the Northwest region of China, analyzed the statistical distribution of allele frequency at 9 STR loci, and attempted to reconstruct the genetic structure and reveal the respective gene flows. Our analyses also consider geographic and linguistic factors. With these factors in mind, we have quantitatively analyzed the variance components contributed by genetic differentiation, geographic isolation and linguistic differences.

## Materials and Methods

### 1 Samples and population data

We obtained samples of Han individuals residing in Xi'an (N = 84); Hui residing in Ningxia (N = 82); Mongol residing in Inner-Mongolia (N = 94); Uyghur (N = 88), Kazakh (N = 100), Kirghiz (N = 101), and Uzbek (N = 58) who live in Sinkiang Uighur Autonomous Region; Salar (N = 100), Yugur (N = 120) and Baoan (N = 120) who live in Gansu; and Dongxiang (N = 118) and Tu (N = 102) who reside in Qinghai (Table 1). All individuals were selected randomly with the appropriate informed consent. Confirmation was obtained that all four grandparents of each genotyped individual had been born in the same area. The sample size used is sufficient for a genetic population analysis using microsatellites [18]. Furthermore, as the allele frequencies of Kazakh, Salar, Tu and Baoan have been published previously by our lab [19]–[21], we adopted the original data instead of repeating the experiment. In addition, the population data for the Han living in Sinkiang Uighur Autonomous Region were acquired from one Chinese study [22].

**Table 1:** Name of the studied populations, number of chromosomes, geographic coordinates, linguistic background, religion and reference.

Sample	chromosome	Geographic coordinates		Linguistic affiliation	Religion	Reference
	number	longitude	latitude			
Han_XA^a^	168	108.57	34.16	Sino-Tibetan	No specific	
Hui	164	106.28	38.02	Sino-Tibetan	Islamic	
Mongol	188	111.67	40.92	Altaic, Mongolian	Lamaism^b^	
Uyghur	176	76.50	37.60	Altaic, Turkic	Islamic	
Kazakh	200	82.50	43.50	Altaic, Turkic	Islamic	[19]
Uzbek	116	81.20	43.55	Altaic, Turkic	Islamic	
Kirghiz	201	75.43	40.33	Altaic, Turkic	Islamic	
Salar	200	102.28	35.50	Altaic, Turkic	Islamic	[20]
Tu	204	101.57	36.50	Altaic, Mongolian	Lamaism	[20]
Dongxiang	236	102.70	35.80	Altaic, Mongolian	Islamic	
Yugur	240	100.50	37.20	Altaic, Mongolian	Islamic	
Baoan	240	100.16	33.10	Altaic, Mongolian	Islamic	[21]
Han_XJ	300	87.38	43.92	Sino-Tibetan	No specific	[23]

a : Han_XA represents the Han population living in Xi'an, while Han_XJ represents the Han population in Sinkiang Uighur Autonomous Region.

b : “Lamaism” is a branch of Buddhism that is popular in some regions of China, especially in West China.

The study was approved by the Xi'an Jiaotong University Ethics Committee. All participants signed the written informed consent. One of previous study was published using part of these samples [23].

### 2 DNA extraction and genotyping

Genomic DNA was extracted using the Chelex-100 protocol as described by Walsh et al. and quantified spectrophotometrically[24]. Multiplex PCR amplification was performed on approximately 1–3 ng of genomic DNA in a total reaction volume of 25 µl, consisting of 9.5 µl of the AmpFlSTR Identifiler PCR reaction mix, 0.5 µl of AmpliTaq Gold DNA polymerase, and 5.0 µl of the AmpFlSTRI dentifiler primer set. Amplification was carried out in a 9700 Perkin-Elmer DNA Thermal Cycler (Applied Biosystems) using 28 cycles under the following conditions (after an initial denaturation step of 11 min at 95°C): 94°C for 1 min, 59°C for 1 min, 72°C for 1 min (following the recommendations from the AmpFlSTR Identifiler PCR kit manufacturer's manual). The amplified DNA products were separated and detected using an ABI Prism 3730 DNA sequencer (Applied Biosystems). One microliter of PCR product was combined with 12 µl of formamide and 0.5 µl of size standard (GeneScan 500 LIZ). The resultant data analysis and allele designation were carried out using the GeneScan and Genotype software programs.

### 3 Data analysis

Allele frequencies were estimated by gene counting following exact tests of Hardy-Weinberg equilibrium with Genepop[25]. Gene diversity was estimated as n/(n-1)(1-∑*x*_i_^2^), where *x* is the estimated frequency of the *i*th allele in the system. The combined power of exclusion probability of paternity (EPP) and combined probability of matching(PM) for the nine STR systems for each population were calculated as 1-(1-EPP_1_)*(1-EPP_2_)…*(1-EPP_9_) and PM_1_*PM_2_*…*PM_9_, respectively, where EPP_n_ and PM_n_ can be estimated by the Powerstats program [26].

Nei's D_A_ distance was selected to be the genetic measure, as it best reflects the real differentiation among populations; it was calculated by the Dispan program [27], [28]. A neighbor-joining phylogenetic tree based on genetic distance (with bootstrap 1000 times) was constructed by Mega 4.1. Because the matrix of allele frequencies of several STR loci has some defects [29], we transformed it into its variance-covariance matrix by PAST[30]. SPSS 13.0 was then used to perform the principal component analysis and draw the scatter plot. The R matrix model of Harpending and Ward was applied to perform the regression analysis with the formula 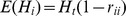, where r_ii_ is the genetic distance of a particular population from the gene frequency centroid, which can be calculated from allele frequency data, as in the formula 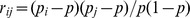. H_i_ is the average heterozygosity of the *i*th population, and H_t_ is equal to the overall mean heterozygosity of the entire population [31].

The geographic distances were entered as a matrix of the great-circle distances between pairs of populations and were assessed on the basis of population geographic coordinates [32]. Linguistic distances were estimated as simple dissimilarity indexes ranging from 0 to 4. Languages belonging to different phyla were assigned a value of 4; languages belonging to different branches, 3; languages belonging to different families, 2; different languages, 1; and the same language, 0 [33]. The linguistic classification of the Northwest China languages used in this process was adopted from the Ethnologue online language database (http://www.ethnologue.com).

## Results

### 1 Gene diversity and forensic parameters

Table 2 summarizes the genetic polymorphisms of the selected 13 populations. The gene diversity values across the 9 STR loci are all above 0.7 (with a range of 0.7435∼0.7793). The Hui have the lowest gene diversity value and the Kirghiz the highest value. On the other hand, the total number of alleles detected is generally greater than 60, while the Han in Xi'an and the Hui have the lowest number at 63, and the Tu have the highest number at 80. The overall Gst value for all loci is 0.0142. The combined EPP is always used to estimate the application value for a given marker system, and the combined probability of matching is always considered an important index in the individual identification or discrimination. These two values together show great value in the application of the 9 STR marker system for the 13 selected populations. The EPP value in all cases is above 0.9999, and the EPM value is below 10^−8^. Furthermore, more than 500 paternity cases and cases of individual identification have been successfully resolved using the 16 Powerplex system, which contains the abovementioned 9 STR loci.

**Table 2:** Genetic polymorphism of 13 populations for the 9 STR system.

populations				
	gene diversity	allele number	Combined EPP	Combined PM
Han_XA	0.7500	63	0.99990	3.070*10^−9^
Han_XJ	0.7584	73	0.99980	1.050*10^−9^
Hui	0.7435	63	0.99975	1.520*10^−8^
Uyghur	0.7722	67	0.99987	1.250*10^−9^
Kazakh	0.7651	69	0.99998	0.308*10^−9^
Kirghiz	0.7793	70	0.99999	0.128*10^−9^
Dongxiang	0.7547	74	0.99994	1.085*10^−9^
Tu	0.7619	80	0.99996	0.720*10^−9^
Uzbek	0.7732	65	1.00000	0.228*10^−9^
Salar	0.7532	67	0.99994	0.866*10^−9^
Baoan	0.7621	67	0.99985	1.130*10^−9^
Yugur	0.7615	66	0.99996	1.250*10^−9^
Mongol	0.7662	69	0.99994	2.060*10^−9^

### 2 Genetic distance and phylogenetic trees

**Figure 2:**
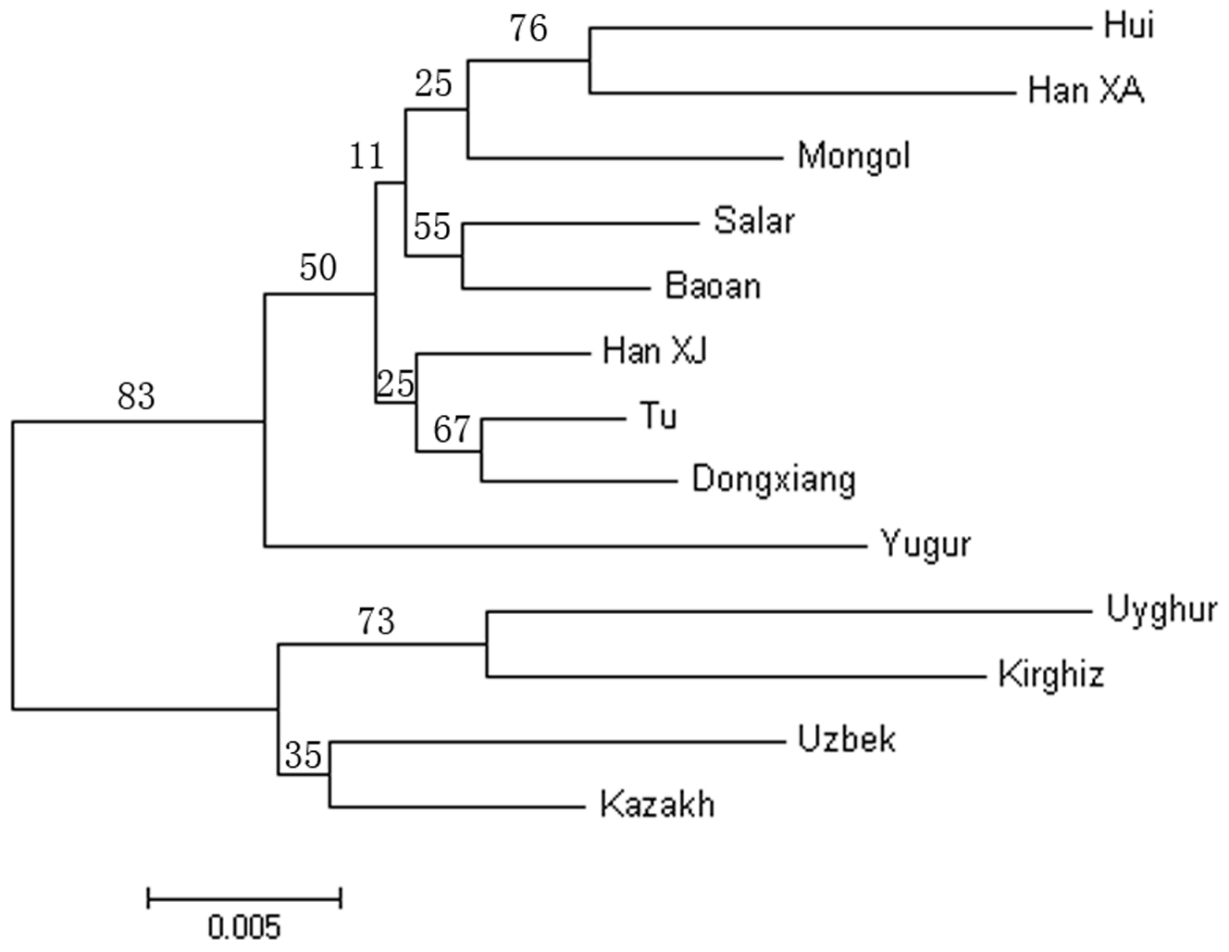
Neighbor-joining phylogenetic tree based on D_A_ distance.

Pairwise genetic distance were shown in Table 3. Among populations from Northwest China, the largest value was found between the Kazakh and Han_XA samples, with a pairwise distance of 0.06, suggesting a relatively remote relationship. A neighbor-joining tree for the 13 population samples was constructed using the pairwise D_A_ distance with 1000 bootstrap times (Table 3, Figure 2). All of the populations were clustered into two main branches (83% bootstrap support). One included four populations in Sinkiang Uighur Autonomous Region, while the other nine populations were grouped together. Conversely, the closest relationship was between the Tu and Dongxiang, with the lowest distance at only 0.0092 (67% bootstrap support). Other main sub-clusters with >50% bootstrapping support included Uyghur and Kirghiz (73%), Salar and Baoan (55%).

**Table 3:** Pairwise D_A_ distance among the 13 populations.

	Uygur	Uzbik	Kirgiz	Kazak	Sala	Tu	Dongxiang	Yugu	Baoan	Mongol	Hui	Han_XJ	Han_XA
Uygur													
Uzbik	0.0398												
Kirgiz	0.0291	0.0271											
Kazak	0.0305	0.0188	0.0256										
Sala	0.0477	0.0426	0.0411	0.0331									
Tu	0.0508	0.0324	0.0419	0.0315	0.0136								
Dongxiang	0.0483	0.0377	0.0480	0.0351	0.0172	0.0092							
Yugu	0.0496	0.0396	0.0509	0.0390	0.0293	0.0221	0.0244						
Baoan	0.0380	0.0363	0.0403	0.0301	0.0114	0.0150	0.0164	0.0256					
Mongol	0.0427	0.0385	0.0402	0.0314	0.0156	0.0179	0.0204	0.0328	0.0164				
Hui	0.0510	0.0519	0.0548	0.0395	0.0265	0.0284	0.0254	0.0383	0.0257	0.0247			
Han_XJ	0.0476	0.0357	0.0407	0.0327	0.0138	0.0102	0.0116	0.0240	0.0134	0.0180	0.0223		
Han_XA	0.0554	0.0188	0.0500	0.0600	0.0446	0.0265	0.0243	0.0208	0.0361	0.0226	0.0230	0.0245	

### 3 Principal component analyses

**Figure 3:**
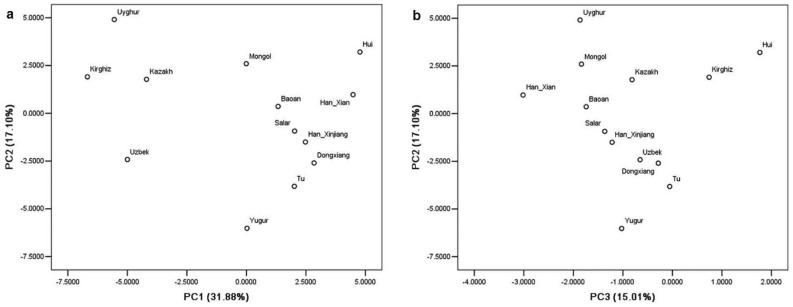
Plot of principal component analysis. Note:a. Scatter plot of the first two principal components (PC1, PC2). b. Scatter plot of the PC2 and PC3

The gene frequency matrix is characterized by closed datawith aneffect of closure, which confounds the analysis of the population genetic structure, we have used the model established by Xue et al.[29] to perform the principal component analysis, which uses the averaged covariance matrix calculated from gene frequencies. The three main principal components from the result have a ratio of variance of 31.88%, 17.1%, and 15.01% respectively, with the total ratio at 63.99%. The two-dimensional scatter plot (Figure 3) revealed that four populations in Sinkiang Uighur Autonomous Region (Uyghur, Kirghiz, Uzbek and Kazakh) are apparently separated from the other populations by the first principal component (Figure 3a).

### 4 R matrix analyses

**Figure 4:**
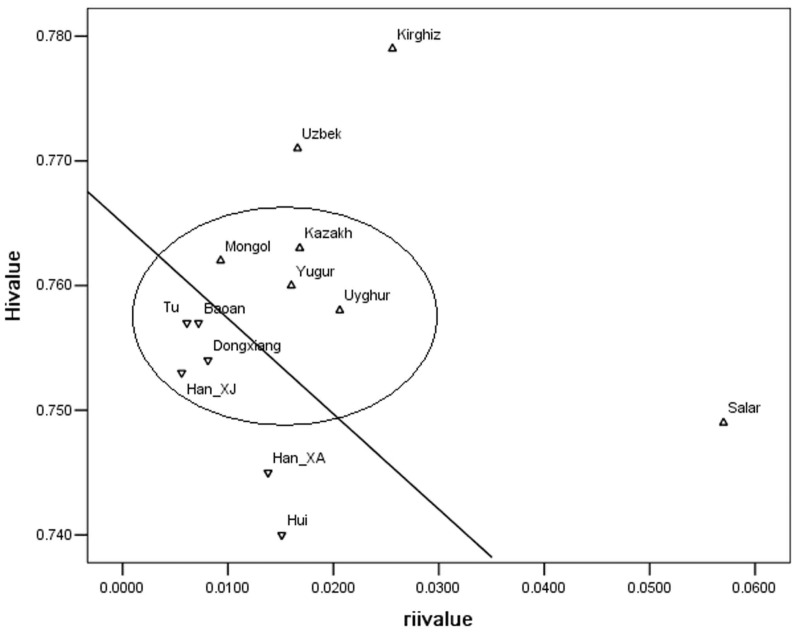
Regression plots of 13 populations.

A regression plot was built to examine the level of genetic exchange and patterns of gene flow within the general region of Northwest China using the R matrix model described by Reddy et al. [34]. As shown in Figure 4, the Kirghiz, Uzbek and Salar populations have received a higher-than-normal level of gene flow from outside because they fall far above the expected regression line. In comparison, the more isolated populations include the Hui and Han in Xi'an, which fall far below the line. The remaining eight populations are clustered into one group that are scattered on either side, but close to the regression line, indicating they received an average level of gene flow in the total region.

### 5 Mantel tests

The results of the Mantel tests are shown in Table 4 and include correlation and partial correlation for three distance matrices. We performed the analysis for all 13 populations. The Dgen (genetic distance) and Dgeo (geographic distance) correlation shows a significant *P* value (0.002), with a correlation coefficient of 0.4769 and a 21.58% variance. In contrast, there is no significant difference for Dgen and Dlan, with a low correlation coefficient and variance. When linguistic is kept constant, the partial correlation coefficient for genetics and geography is 0.4516, with high statistical significance (*P*  = 0.004); conversely, the correlation coefficient is not significant for genetics and language, with a *P* value of 0.096 and a correlation coefficient value of 0.1230.

**Table 4:** Mantel tests of three different types of distances.

	Mantel test		
Matrices compared	Correlation coefficient	P value	Variance explained
Dgen and Dgeo	0.4769	0.002	0.2158
Dgen and Dlan	0.2100	0.024	0.0233
Dgen and Dgeo, Dlan constant	0.4516	0.004	
Dgen and Dlan, Dgeo constant	0.1230	0.096	

Note: Dgen stands for genetic distance, Dgeo as geographic distance, and Dlan language distance.

## Discussion

The Northwest region of China was the starting point of the ancient “Silk Road” and served to link Central China in the East to Central Asia, South Asia and even Europe in the West. According to historical records, cultural and commercial communication between the eastern and western was frequent in this region. Moreover, inter-population marriage and genetic exchange among the different populations were very common. It is now quite clear that the Uyghur, Uzbek, Kazakh and Kirghiz populations, which originally lived in Central Asia, migrated into the Sinkiang Uighur Autonomous Region in China in approximately the 5th century, A.D. This is supported by genetic distance we calculated using 13 Northwest Chinese populations and 4 other world populations (Table S1) [35]–[37]. Apparently those 4 minority populations from Sinkiang are closer to Turkish or Caucasian American, but more distance from Japanese.

There is still no definitive answer regarding the origin of the five ethnic populations living in the Gansu and Qinghai Provinces. Historical records support two hypotheses about the origin of the Tu population. The first and more popular hypothesis proposes that their ancestors were actually from Liaoning Province in the East and that they later migrated into Qinghai and Gansu Provinces in the early 4th century and inter-married with local Mongolian, Tibetan and Han populations [3], [4], [38]. The second hypothesis considers the Tu population to be descendants of 13th century Mongolian soldiers and women from local nomadic groups [2], [3], [39]. Although controversy exists, there is no doubt that extensive genetic admixture once occurred in the history of the Tu population. The ancestors of the Baoan population are most likely Mongolians who arrived with the Turkistan soldiers after the 13th century. These people first reclaimed and grazed their cattle along the “Tongren region” in Qinghai Province and gradually formed a new ethnic population after long-time fusion and inter-marriage with the local Hui, Dongxiang, Salar, Tibetan and Han populations [2]–[4], [34]. The origin of the Yugur population may date back to the ancient “Uighur population” that established the “Uighur Kingdom” in 745 AD, covering the grasslands south of Lake Baikal, north of Yinshan Mountain, west of Khingan Mountains and east of Altai Mountain [39]. There is also controversy concerning the origin of the Dongxiang population. One hypothesis suggests that the ancient Hui population living in Dongxiang, together with local Mongolian, Han and Tibetan populations, inter-married and formed the current Dongxiang population. A second hypothesis argues for a Mongolian origin [2]–[4]. The Salar population is derived from the Ogus group from the Western Turkic State, which first lived in China and later migrated to the central Asian region. In the 13th century, the Ogus migrated through the Samarkand region to east Qinghai Province, where they settled. They gradually adapted to the new environment and inter-married with local Han, Tibetan, Hui and Mongolian populations, finally forming the current Salar population [2]–[4].

The polymorphisms of the nine selected autosomal microsatellite markers have been reported in different populations of the world [40]. Their application as CODIS markers for personal discrimination and human identification has been evaluated, and it was demonstrated that the use of these forensically accepted loci with high heterozygosity and allele numbers is feasible for the study of population differentiation and admixture. The principal component analysis extracted several PCs as new variables by the dimension reduction method, which can be used to determine the features of and basic reasons for population differentiation. Complementary phylogenetic trees constructed from specific genetic distances are ideal tools to deduce the evolutionary relationships and origins of different populations[41]. We applied these two major statistical approaches to the datasets and found that minorities that live in Sinkiang Uighur Autonomous Region tend to be more differentiated than other populations. Two major elements should be taken into consideration when drawing any conclusions regarding the patterns of gene flow for the 13 populations. One is the demographic size of the population, and the other is the equilibrium of genetic drift and population migration [42]. Therefore, the Han in Xi'an and the Hui did not receive an average level of gene flow based on the R-matrix analysis might be a result of the large demographic size involved. In other words, marriage between individuals from these two populations with members of other populations would be diluted and have little effect, especially because the majority of marriages were within the population.

Populations from different continents that are geographically close are also more similar genetically than predicted by the simple hypothesis that they are from their respective continents [43], [44]. Recent studies have analyzed the origin and evolutionary relationship of different major world populations and have attempted to explain the genetic variance by geographic and linguistic characteristics using large scale genetic markers. Most of these published papers considered geography to be the main factor and argued that language exerted a secondary but detectable effect [32], [33], [44], [45]. For populations that are geographically close, genetic and geographic distances are often highly correlated. QasimAyub et al. [44] has suggested that the genetic relationships of the 19 extant human populations around the world, as ascertained by 182 microsatellites, are dictated primarily by geographic proximity, with R = 0.484 (p = 0.05). In a subsequent paper, Elise M. S. Belle et al. [33] pointed out that the genetic differences of 52 world-wide populations, indicated by 377 microsatellites, appear to more closely reflect geographic differentiation, although linguistic differences also have a detectable effect on DNA diversity. This latter article first quantified the contributions of geography and language to the populations living in Northwest China, avoiding purely subjective conclusions. Partial correlation from the Mantel test for 13 independent populations suggested that geographical differences have a significant influence on genetic differentiation (r of partial correlation equals 0.4516, while the *P* value is 0.0004.). Language distance represents an additional contribution to the effect (correlation coefficient at 0.123 with *P* value 0.096). In our current analysis, we have considered only the geographic coordinates for calculating the geographical distance but have not included the complicated terrain of the Northwest, which is characterized by mountains, deserts and plateaus. In the future, we will establish a more complex and precise mathematical model to quantify the geographic isolation.

In conclusion, our results demonstrate that high–level admixture does exist in the Northwest region of China, which is part of the Silk Road of ancient times. However, the populations living in northern China in Sinkiang Uighur Autonomous Region, which include Uyghur, Kazakh, Uzbek and Kirghiz, are closely clustered, but quite distant from other populations living in Qinghai and Gansu and from the subpopulations of the Han, Hui and Mongol. Those findings reveal that geographic isolation plays a significant role in population differentiation, whereas language differences exert a much smaller influence.

## Supporting Information

Table S1 **Pairwise D_A_ distance between the 17 populations.**
(DOCX)
