# Correction: The Contribution of Genetic Diversity to Subdivide Populations Living in the Silk Road of China

**DOI:** 10.1371/journal.pone.0105847

**Published:** 2014-08-12

**Authors:** The PLOS ONE Staff

There are errors in the author affiliations. The affiliations should appear as shown here:

Zhe Zhang ^1,2,3^; Shuguang Wei ^2^; Hongsheng Gui ^2^; Zuyi Yuan ^1,3^; Shengbin Li ^1,2^

1 Key Laboratory of Environment and Gene Related to Diseases, Ministry of Education, College of Medicine, Xi’an Jiaotong University, Shaanxi, China

2 The Key Laboratory of National Ministry of Health for Forensic Sciences, College of Medicine and Forensics, Xi’an Jiaotong University, Shaanxi, China

3 Department of Cardiovascular Medicine, First Affiliated Hospital of Medical College, Xi'an Jiaotong University, Shaanxi, China.
