# Supplementary material for: The Contribution of Genetic Diversity to Subdivide Populations Living in the Silk Road of China
Source: PLoS One. 2014 May 14;9(5):e97344. doi: 10.1371/journal.pone.0097344 (PMC4020837; doi:10.1371/journal.pone.0097344)
Supplement: Table S1 — Pairwise DA distance between the 17 populations. (DOCX) [file pone.0097344.s001.docx]

**Table S1. Pairwise D_A_ distance between the 17 populations**

|  | Uyhgur | Uzbek | Kirghiz | Kazakh | Salar | Tu | Dongxiang | Yugur | Baoan | Mongol | Hui | Han_XJ | Han_XA |
| --- | --- | --- | --- | --- | --- | --- | --- | --- | --- | --- | --- | --- | --- |
| Uyghur |  |  |  |  |  |  |  |  |  |  |  |  |  |
| Uzbek | 0.0398 |  |  |  |  |  |  |  |  |  |  |  |  |
| Kirghiz | 0.0291 | 0.0271 |  |  |  |  |  |  |  |  |  |  |  |
| Kazakh | 0.0305 | 0.0188 | 0.0256 |  |  |  |  |  |  |  |  |  |  |
| Salar | 0.0477 | 0.0426 | 0.0411 | 0.0331 |  |  |  |  |  |  |  |  |  |
| Tu | 0.0508 | 0.0324 | 0.0419 | 0.0315 | 0.0136 |  |  |  |  |  |  |  |  |
| Dongxiang | 0.0483 | 0.0377 | 0.048 | 0.0351 | 0.0172 | 0.0092 |  |  |  |  |  |  |  |
| Yugur | 0.0496 | 0.0396 | 0.0509 | 0.039 | 0.0293 | 0.0221 | 0.0244 |  |  |  |  |  |  |
| Baoan | 0.038 | 0.0363 | 0.0403 | 0.0301 | 0.0114 | 0.015 | 0.0164 | 0.0256 |  |  |  |  |  |
| Mongol | 0.0427 | 0.0385 | 0.0402 | 0.0314 | 0.0156 | 0.0179 | 0.0204 | 0.0328 | 0.0164 |  |  |  |  |
| Hui | 0.051 | 0.0519 | 0.0548 | 0.0395 | 0.0265 | 0.0284 | 0.0254 | 0.0383 | 0.0257 | 0.0247 |  |  |  |
| Han_XJ | 0.0476 | 0.0357 | 0.0407 | 0.0327 | 0.0138 | 0.0102 | 0.0116 | 0.024 | 0.0134 | 0.018 | 0.0223 |  |  |
| Han_XA | 0.0554 | 0.0188 | 0.05 | 0.06 | 0.0446 | 0.0265 | 0.0243 | 0.0208 | 0.0361 | 0.0226 | 0.023 | 0.0245 |  |
| Japanese[[1](#_ENREF_1)] | 0.0511 | 0.0517 | 0.0804 | 0.0762 | 0.0403 | 0.0324 | 0.0317 | 0.0584 | 0.0387 | 0.0359 | 0.0515 | 0.0324 | 0.0298 |
| Turkish[[2](#_ENREF_2)] | 0.0230 | 0.0422 | 0.0470 | 0.0470 | 0.0697 | 0.0711 | 0.0698 | 0.0730 | 0.0872 | 0.0623 | 0.1047 | 0.0889 | 0.0744 |
| Cau_American[[3](#_ENREF_3)] | 0.0399 | 0.0621 | 0.0633 | 0.0647 | 0.1102 | 0.1087 | 0.1100 | 0.1173 | 0.1253 | 0.0940 | 0.1428 | 0.1334 | 0.1122 |
| Afr_American[[3](#_ENREF_3)] | 0.1013 | 0.1051 | 0.1006 | 0.1306 | 0.1265 | 0.1577 | 0.1420 | 0.1448 | 0.1728 | 0.1275 | 0.1703 | 0.1788 | 0.1624 |

References

1. Budowle B, Shea B, Niezgoda S, Chakraborty R (2001) CODIS STR loci data from 41 sample populations. Journal of Forensic Sciences 46: 453-489.

2. Akbasak BS, Budowle B, Reeder DJ, Redman J, Kline MC (2001) Turkish population data with the CODIS multiplex short tandem repeat loci. Forensic science international 123: 227-229.

3. Budowle B, Moretti TR, Baumstark AL, Defenbaugh DA, Keys KM (1999) Population data on the thirteen CODIS core short tandem repeat loci in African-Americans, US Caucasians, Hispanics, Bahamians, Jamaicans, and Trinidadians. Journal of forensic sciences 44: 1277-1286.
